# Physical aspects of epithelial cell–cell interactions: hidden system complexities

**DOI:** 10.1007/s00249-024-01721-z

**Published:** 2024-09-10

**Authors:** Ivana Pajic-Lijakovic, Milan Milivojevic, Peter V. E. McClintock

**Affiliations:** 1 Faculty of Technology and Metallurgy, Department of Chemical Engineering, University of Belgrade, Belgrade, Serbia; 2 Department of Physics, Lancaster University, Lancaster, LA1 4YB UK

**Keywords:** Epithelial monolayers, Viscoelasticity, Focal adhesions, Adherens junctions, Cell mechanical stress, Cell alignment

## Abstract

The maintenance of homeostasis and the retention of ordered epithelial cell self-organization are essential for morphogenesis, wound healing, and the spread of cancer across the epithelium. However, cell–cell interactions in an overcrowded environment introduce a diversity of complications. Such interactions arise from an interplay between the cell compressive and shear stress components that accompany increased cell packing density. They can lead to various kinds of cell rearrangement such as: the epithelial-to-mesenchymal cell state transition; live cell extrusion; and cell jamming. All of these scenarios of cell rearrangement under mechanical stress relate to changes in the strengths of the cell–cell and cell–matrix adhesion contacts. The objective of this review study is twofold: first, to provide a comprehensive summary of the biological and physical factors influencing the effects of cell mechanical stress on cell–cell interactions, and the consequences of these interactions for the status of cell–cell and cell–matrix adhesion contacts; and secondly, to offer a bio-physical/mathematical analysis of the aforementioned biological aspects. By presenting these two approaches in conjunction, we seek to highlight the intricate nature of biological systems, which manifests in the form of complex bio-physical/mathematical equations. Furthermore, the juxtaposition of these apparently disparate approaches underscores the importance of conducting experiments to determine the multitude of parameters that contribute to the development of these intricate bio-physical/mathematical models.

## Introduction

The reorganization of tissues, which is crucial for biological processes like morphogenesis, wound healing, and cancer progression, is driven primarily by the mechanical interactions between cells during collective cell migration (Barriga and Mayor 2019; Alert and Trapet, 2020). In order to maintain cellular homeostasis, it is vital to maintain and renew the adhesion contacts between cells and the extracellular matrix (Eisenhoffer et al. 2012; Roycroft and Mayor 2016). This enables cells to retain their connections with neighbouring cells as well as with the surrounding matrix, thereby facilitating coordinated migration (Shellard and Mayor 2020).

Collective cell migration during organ formation, wound healing and cancer metastasis involves tissue deformation, which leads in turn to the generation of cell mechanical stress (Pajic-Lijakovic and Milivojevic 2020, 2022a). The mechanical stress consists of normal (compression or tensional) and shear stress components. All stress components have been measured within migrating epithelial monolayers (Serra-Picamal et al. 2012; Tambe et al., 2013; Notbohm et al. 2016; Pérez-González et al. 2019). Strong cell–cell and cell–matrix adhesion complexes ensure an accumulation of mechanical stress rather than its dissipation (Pajic-Lijakovic and Milivojevic 2022a). However, an increase in the mechanical stress influences cell–cell interactions, which have a feedback impact on the state of adhesion complexes (Eisenhoffer et al. 2012; Iyer et al. 2019; Pajic-Lijakovic and Milivojevic 2022a). The physical mechanisms underlying the dynamic regulation of cell adhesions under mechanical stress are not yet understood. The cause–consequence relationships between the strength of the adhesion complexes, the cell–cell interactions, and the cell mechanical stress influence the cell rearrangement.

Epithelial cells undergo a variety of cellular processes when cell–cell and/or cell–matrix adhesion contacts are perturbed under higher compressive and shear stress components. These processes include the epithelial-to-mesenchymal transition (EMT), live cell extrusion, and the cell-jamming state transition. Cell mechanical stress triggers complex biochemical processes leading to loss of E-cadherin, the main component of epithelial adherens junctions, which is a prerequisite of the EMT (Le Bras et al. 2012; Yang et al. 2020). Epithelial cells are commonly identified by their cuboidal morphology, restricted cell movement, apical-basal polarity, and robust cell–cell adhesions mediated by E-cadherin. Conversely, mesenchymal-like cells display an elongated cellular structure, heightened migratory capacity, front-rear cell polarity, and less-pronounced cell–cell adhesion mediated by N-cadherin (Gandalovičová et al. 2016). However, when subjected to increased compressive stress, epithelial cells experience a loss of adhesion with the surrounding matrix, resulting in live cell extrusion. Additionally, cell jamming can also occur under higher compressive stress, leading to cell repolarisation and a weakening of both cell–cell and cell–matrix adhesion. This transition causes cells to shift from a contractile to a noncontractile state. On the other hand, during the processes of epithelial–mesenchymal transition (EMT) and live cell extrusion, cells retain their epithelial phenotype and maintain an active contractile state. Ultimately, all these of cellular processes contribute to a reduction in mechanical stress and energy dissipation within the cell. Given the occurrence of all of these cellular processes under high cell compressive stress, it becomes imperative to explore the role of cell shear stress as the primary physical factor responsible for the generation of diverse cell rearrangement scenarios, which is the goal of this theoretical consideration.

To gain a comprehensive understanding of the connections between the different scenarios of cell rearrangement under mechanical stress and the disruption of cell–cell and cell–matrix adhesion contacts, it is essential to explore the diverse physical aspects of cell–cell interactions. Within the realm of collective cell migration, two interdependent types of cell–cell interaction are examined: positional interactions and orientational interactions (Alert and Trapet, 2020). The orientational interactions between cells encompass head-on interactions and cell glancing interactions. On the other hand, positional interactions between cells can either be attractive or repulsive, depending on the distance between the cells.

Although the concept of cell jamming has been discussed primarily in relation to head-on interactions that lead to the contact inhibition of locomotion (Garcia et al. 2015; Pajic-Lijakovic and Milivojevic 2023a), the emergence of live cell extrusion is instead induced by intense cell glancing interactions occurring within the cell regions that contain topological defects of cell alignment (Saw et al. 2017). Head-on interactions between cells trigger a process known as cell repolarisation, which is accompanied by a weakening of the adhesive contacts between cells and the extracellular matrix (Roycroft and Mayor 2016). When the time interval between two consecutive cell–cell collisions is shorter than the repolarization time, the cells undergo a transition into a jammed state (Pajic-Lijakovic and Milivojevic 2022a, 2023a). In contrast to head-on interactions, cell glancing interactions are insufficiently strong to induce cell repolarisation (Lin et al. 2018). As a result, cells maintain strong adhesive contacts with neighbouring cells. These interactions could cause the cells to undergo self-rotation during realignment, leading to the generation of torsional shear stress within the cell–matrix interface. This shear stress ultimately disrupts the adhesive contacts between the cells and the extracellular matrix, potentially resulting in cell extrusion (Paddillaya et al., 2019). The disruption of cell–cell adhesion contacts, which is a prerequisite for the epithelial-to-mesenchymal transition, can be induced by the application of shear stress to the cells.

The primary objectives of the theoretical analysis that follows are: (1) to explore the impact of cell–cell interactions on the integrity of cell–cell and cell–matrix adhesion contacts; (2) to point out the physical aspects of cell–cell interactions; (3) to highlight the role of cell mechanical stress in promoting cell–cell interactions; and (4) to examine the factors leading to the development of cell mechanical stress during the collective migration of epithelial monolayers on substrate matrices. We emphasize the relationship between the strengths of the cell–cell and cell–matrix adhesion contacts, of the cell–cell interactions, and the generation of cell mechanical stress, by presenting a new multiscale biophysical model developed by combining some old and new physical models. Insights gained in this work will serve to increase our understanding of the maintenance of epithelial homeostasis which underlies its normal barrier function in each organ system.

## Scenarios of cell rearrangement caused by cell–cell interactions in an overcrowded environment

Compressive and shear components of mechanical stress have been recognised as the one of main physical factors, which induce the various scenarios of cell rearrangement, such as the epithelial-to-mesenchymal transition (EMT), live cell extrusion, and the jamming state transition by influencing cell–cell interactions (Eisenhoffer et al. 2012; Tse et al. 2012; Rizvi et al., 2013; Garcia et al. 2015; Saw et al. 2017; Pajic-Lijakovic and Milivojevic 2023a). Cell–cell interactions perturb the state of adherens junctions and focal adhesions. These perturbations occur due to the interactions between cells in an overcrowded environment. When the compressive stress on cells increases, the packing density of cells also increases, leading to more intense cell–cell interactions (Pajic-Lijakovic et al. 2024). Additionally, cell shear stress can cause disturbances in cell alignment and also the swirling motion of cells (Pajic-Lijakovic and Milivojevic 2022b). As a result, the mechanical stress experienced by cells during collective cell migration plays a crucial role in the emergence of the different cell rearrangement scenarios. In this context, our focus is on the collective migration of epithelial monolayers on substrate matrices.

Collective migration of epithelial monolayers has been considered as active wetting (extension)/de-wetting (compression) (Pérez-González. et al., 2019; Pajic-Lijakovic and Milivojevic 2023b). The phenomenon of wetting describes the ability of a soft matter system to spread across a biointerface in contact with another fluid or solid system, driven by homotypic and heterotypic interactions. The degree of wettability is determined by the relationship between the adhesion and cohesion energies. If the adhesion energy is greater than the cohesion energy, the soft matter system will exhibit wetting (extension); if the cohesion energy is greater, the system will undergo de-wetting (compression). In multicellular systems, the active behavior of cells can promote wetting or de-wetting through coordinated cell movement. The determination of whether cellular systems exhibit wetting or de-wetting behavior hinges on the balance between the adhesion and cohesion energies, a topic that will be explored in greater depth. The key features of cell reorganization during wetting/de-wetting include: (1) non-uniform distribution of the cell adhesion and cohesion energies, as well as of cell velocity, cell packing density, and accumulated cell mechanical stress, and (2) the anisotropic nature of collective cell migration (Serra-Picamal et al 2012; Deforet et al. 2014; Pérez-González. et al., 2019). This phenomenon has been documented in studies by Serra-Picamal et al. (2012), Nnetu et al. (2013), Notbohm et al. (2016), and Tlili et al. (2018). A cell monolayer can be viewed as a collection of interconnected supracellular domains, each characterized by a locally uniform distribution of adhesion and cohesion energies, cell velocity, cell packing density, and accumulated cell mechanical stress, as proposed by Pajic-Lijakovic and Milivojevic (2021a).

**Fig. 1:**
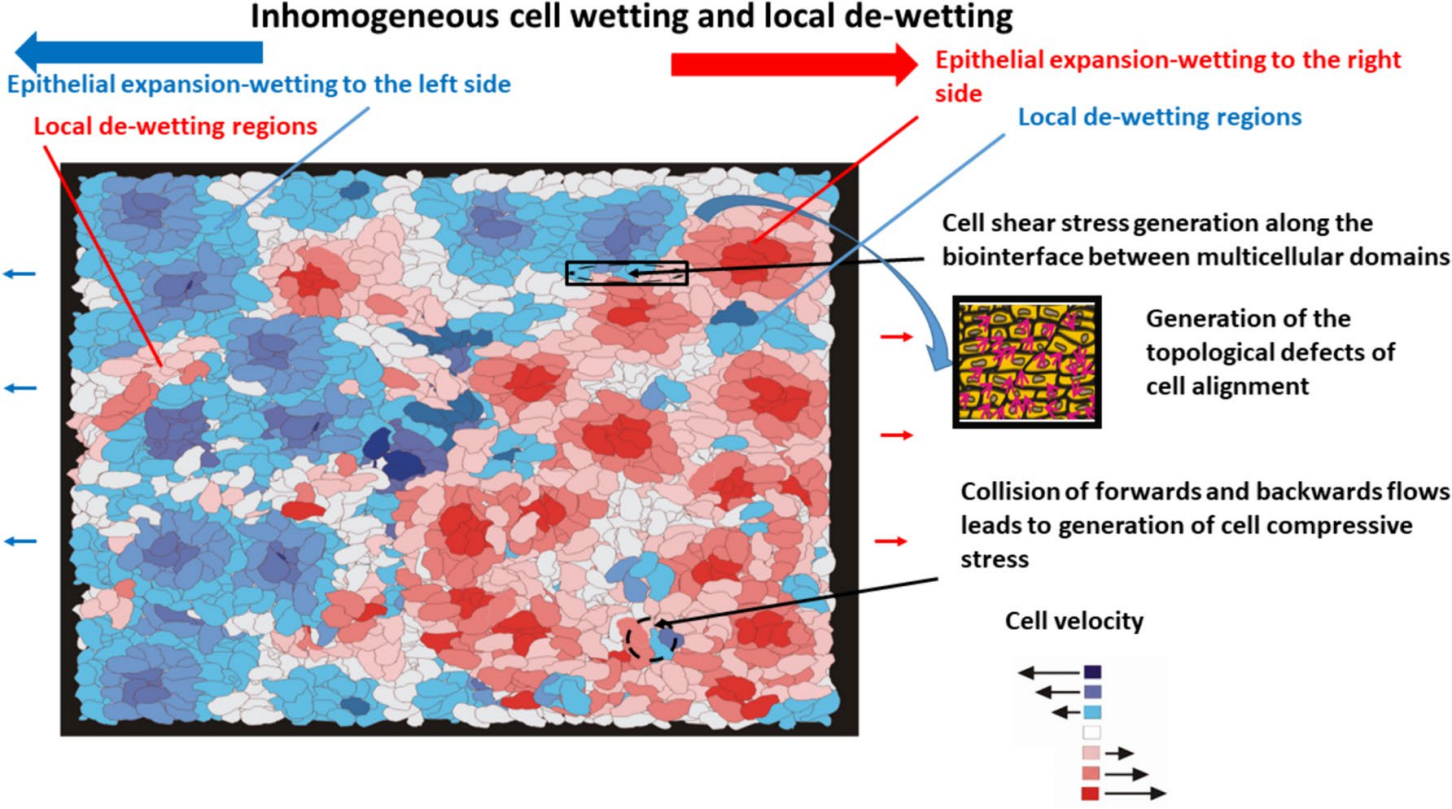
Generation of cell mechanical stress during epithelial active wetting/de-wetting on a substrate matrix characterized by an inhomogeneous distribution of cell velocity and cell mechanical stress (a sketch inspired by the experiments of Serra-Picamal et al. 2012)

Inhomogeneous distribution of cell adhesion and cohesion energy along the cell monolayers causes inhomogeneous wetting/de-wetting as shown in Fig. 1:

Some domains expand more intensively and compress surrounding cell domains (Deforet et al. 2014; Pérez-González. et al., 2019). The extension of domains in the direction of cell movement, i.e. active wetting, causes compression in the perpendicular direction in order to retain structural integrity of the monolayer. Inhomogeneous de-wetting can induce the structural disintegration of the monolayer leading to the formation of holes accompanied by the generation of 3D multicellular aggregates (Douezan and Brochard-Wyart 2012; Deforet et al. 2014; Pérez-González. et al., 2019). Cell shear stress is generated along the biointerface between multicellular domains (Pajic-Lijakovic and Milivojevic 2020). It is in accord with the fact that some domains migrate faster than their surrounding domains (Serra-Picamal et al. 2012; Pérez-González. et al., 2019). In certain instances, the cell monolayer undergoes wetting, while localized areas also experience de-wetting (Serra-Picamal et al. 2012). Consequently, collisions between forwards flows (caused by cell wetting) and backwards flow (caused by cell de-wetting) can result in the generation of cell compressive stress (Pajic-Lijakovic and Milivojevic 2022a, 2023a). Further analysis should focus on highlighting the key characteristics of different cell rearrangement scenarios, which are influenced by the accumulation of mechanical stress within the cells.

### Epithelial-to-mesenchymal transition

During the process of epithelial–mesenchymal transition (EMT), epithelial cells experience a loss of cell–cell adhesion connections and apical-basal polarity (Lamouille et al. 2014; Gandalovičová et al. 2016; Yang et al. 2020). The loss of cell–cell adhesion contacts is related primarily to the endocytosis of E-cadherin (Le Bras et al. 2012). This results in the reorganization of their cytoskeleton, and a shift in the signalling pathways that regulate cell morphology and gene activity, ultimately leading to enhanced cell motility and the acquisition of a mesenchymal phenotype (Lamouille et al. 2014). The epithelial–mesenchymal transition (EMT) process is orchestrated by SNAIL, zinc-finger E-box-binding (ZEB), and basic helix–loop–helix (bHLH) transcription factors, which suppress the expression of epithelial marker genes while promoting the activation of genes linked to the mesenchymal phenotype (Lamouille et al. 2014). Mesenchymal cells establish weak N-cadherin-mediated cell–cell adhesion contacts (Barriga and Mayor 2019). The process of epithelial–mesenchymal transition (EMT) serves as a cellular mechanism for adapting to cell shear stress. It is supported by empirical evidence indicating that cell shear stress promotes the migration of mesenchymal cells while inhibiting the movement of epithelial cells (Riehl et al. 2020).

Fluid shear stress of 0.3 Pa was enough to trigger the EMT in epithelial ovarian cancer (Rizvi et al., 2013). The shear stress caused by active cell wetting corresponds to several tens of Pa (Serra-Picamal et al. 2012; Tambe et al., 2013). The EMT can also be induced by cell compressive stress, although a significantly higher compressive stress is needed. A partial EMT can be caused by an applied compressive stress of ~ 600 Pa (Tse et al. 2012). Furthermore, during the rearrangement of confluent MDCK epithelial monolayers, the maximum cell compressive stress generated was approximately 300 Pa, as reported by Notbohm et al. (2016).

### Live cell extrusion

Cell extrusion occurs in areas where there are irregularities in the alignment of cells within the epithelial monolayers. These topological defects are caused by the combined effects of cell compressive and shear stress components (Saw et al. 2017). In some cases, the shear stress at the biointerface between multicellular regions is not strong enough to break the cell–cell adhesion, but it can perturb cell alignment by promoting glancing interactions between cells. Cell compressive stress in the range of a few hundreds of Pa is enough to induce live cell extrusion (Eisenhoffer et al. 2012).

For cell exclusion to be effective, a number of conditions must be fulfilled: (1) cells must preserve their epithelial phenotype; (2) the target cell must disconnect from the substrate matrix while maintaining its active (contractile) state; and (3) the target cell must be encompassed by contractile cells, resulting in the formation of contractile actin rings (Eisenhoffer et al. 2012). Live cell extrusion, like apoptotic cell extrusion, needs S1P signalling through ROCK-mediated actomyosin contraction (Eisenhoffer et al. 2012). Loss of cell–matrix adhesion contact triggers several signalling pathways, including integrin signalling, PI3K-AkT signalling, and FA signalling, which can lead to cell anoikis, i.e., programmable cell death (Eisenhoffer et al. 2012).

The disturbance of cell alignment causes some cell–cell adhesion contacts to be more stretched then others. The relaxation of these adhesion contacts triggers single cell rotation to facilitate single cell re-alignment. The rotation of the cell results in the generation of torsional stress within the cell matrix, which can lead to the disruption of cell–matrix adhesion contact, all while the cell retains its epithelial phenotype and maintains its active and contractile state. A cell–matrix interfacial shear stress of 4–6 Pa is enough to cause the detachment of an FA (Paddillaya et al., 2019).

### Cell jamming state transition

Cell jamming has been discussed in the context of contact inhibition of locomotion, induced by cell head-on interactions under higher cell compressive stress (Garcia et al. 2015; Pajic-Lijakovic and Milivojevic 2023a). Contact inhibition of locomotion is complex multistep process driven by cytoskeleton rearrangement and dynamics that, in turn, are controlled by the activity of Rho family GTPases (Roycraft and Mayor, 2016).

The compressive stress which favours this type of cell–cell interaction is generated primarily during collisions between forwards and backwards cell flows caused by inhomogeneous cell wetting/de-wetting (Pajic-Lijakovic and Milivojevic 2022a). Cell compressive stress of a few hundred Pa is enough to induce the cell jamming transition (Notbohm et al. 2016). Cell–cell interactions, such as head-on interactions sufficient to induce cell contact inhibition of locomotion accompanied by cell repolarization, play a crucial role in the occurrence of the cell jamming transition (Garcia et al. 2015; Alert and Trepat 2020).

Cell head-on interactions trigger cell repolarisation, which induces migration of cells in the opposite direction (Roycraft and Mayor, 2016). Cell repolarization causes weakening of cell–cell and cell matrix adhesion contacts (Roycraft and Mayor, 2016). The average repolarization time during the rearrangement of confluent epithelial Madin Darby Canine Kidney (MDCK) cell monolayers is 1.28 h (Norbohm et al., 2016). However, intensive cell–cell interactions in an overcrowded environment can extend the repolarisation time, or even block the repolarization process itself. In this case, cells undergo jamming (Garcia et al. 2015; Pajic-Lijakovic and Milivojevic 2022a).

Given that the described scenarios of cell rearrangement occur due to the disruption of cell–cell and cell–matrix adhesion contacts under mechanical stress, it is obviously important to delve into a more comprehensive analysis of the fundamental attributes of these adhesion contacts.

## The main characteristics of cell–cell and cell–matrix adhesion contacts

Epithelial cell–cell adhesion is maintained by three types of junction: tight junctions (zonula occludens), adherens junctions (zonula adherens) and desmosomes (macula adherens) (LeBras et al., 2012). However, collective cell migration is mediated primarily by adherens junctions (AJs) (Barriga and Mayor 2019). The AJs are protein complexes that ensure cell–cell connections within epithelial monolayers via E-cadherin receptors and AJ-associated proteins. Cadherin molecules diffuse laterally through the cell membrane and form clusters (Sumi et al., 2018). Cell–matrix adhesion is established via focal adhesions (FAs). The FAs are protein complexes that ensure cell connection to the substrate matrix via integrin receptors. Integrin molecules, like cadherin molecules, diffuse laterally through the cell membrane and form homotypic protein clusters. The size of a single AJ is $20 \mu m^{2} \le A_{c} \le 100 \mu m^{2}$ (Lin et al. 2018). The FAs are 1–5 µm long and 300–500 nm wide (Legerstee and Houtsmuller 2021). Both types of complexes AJs and FAs are intracellularly linked to the actin cytoskeleton and activate a common set of signalling proteins and actin regulators, such as Rho family GTPases (Mui et al. 2016). Some proteins, like vinculin, represent integral parts of both types of complex (Mui et al. 2016).

The energy of bonds per AJ can be expressed as: $e_{AJ} = N_{cad} \frac{1}{2}k_{AJ} r_{ij}^{2}$ (where N_cad_ is the number of bonded E-cadherins, $k_{AJ}$ is the spring constant of a single bond, and r_ij_ is the average length of an intercellular bond) (Bell et al. 1984). The number of established E- cadherin bonds between neighbouring cells, expressed per single cell, falls in the range of $10 - 10^{3} \frac{bonds}{{\mu m^{2} }}$ (Stirbat et al. 2013). The energy of bonds per FA can be expressed as: $e_{FA} = N_{{\text{int}}} \frac{1}{2}k_{FA} d_{i}^{2}$ (where *N*_int_ is the number of integrin molecules bonded to ligands of substrate matrix, *k*_FA_ is the spring constant of a single bond, and d_i_ is the average length of a cell–matrix bond). Cells are able to self-regulate the strength of FAs and AJs actively by turnover of cadherin and integrin molecules, depending on the magnitude of accumulated cell mechanical stress (Iyer et al. 2019). The turnover time of cadherin and integrin is a few minutes (Lee and Wolgemuth, 2011). Integrin clusters successively attach to, mature, and detach from the substrate matrix during cell movement. The lifetime of FAs is several tens of minutes (Stehbens and Wittmann 2014). This attachment/detachment of FAs during cell movement protects cell against accumulation of cell–matrix shear stress.

Biological processes such as cell signaling and gene expression are included in this regulation of the state of AJs and FAs (Barriga and Mayor 2019). Cell response under extension or compression is mainly regulated by interplay between E-cadherin and integrin levels on the one hand, and distribution of actomyosin on the other, which influence the number of established bonds within both types of complex as well as their strength, conformational changes of cadherin and integrin molecules, and the cortical tension (Iyer et al. 2019; Sumi et al., 2018).

**Fig. 2:**
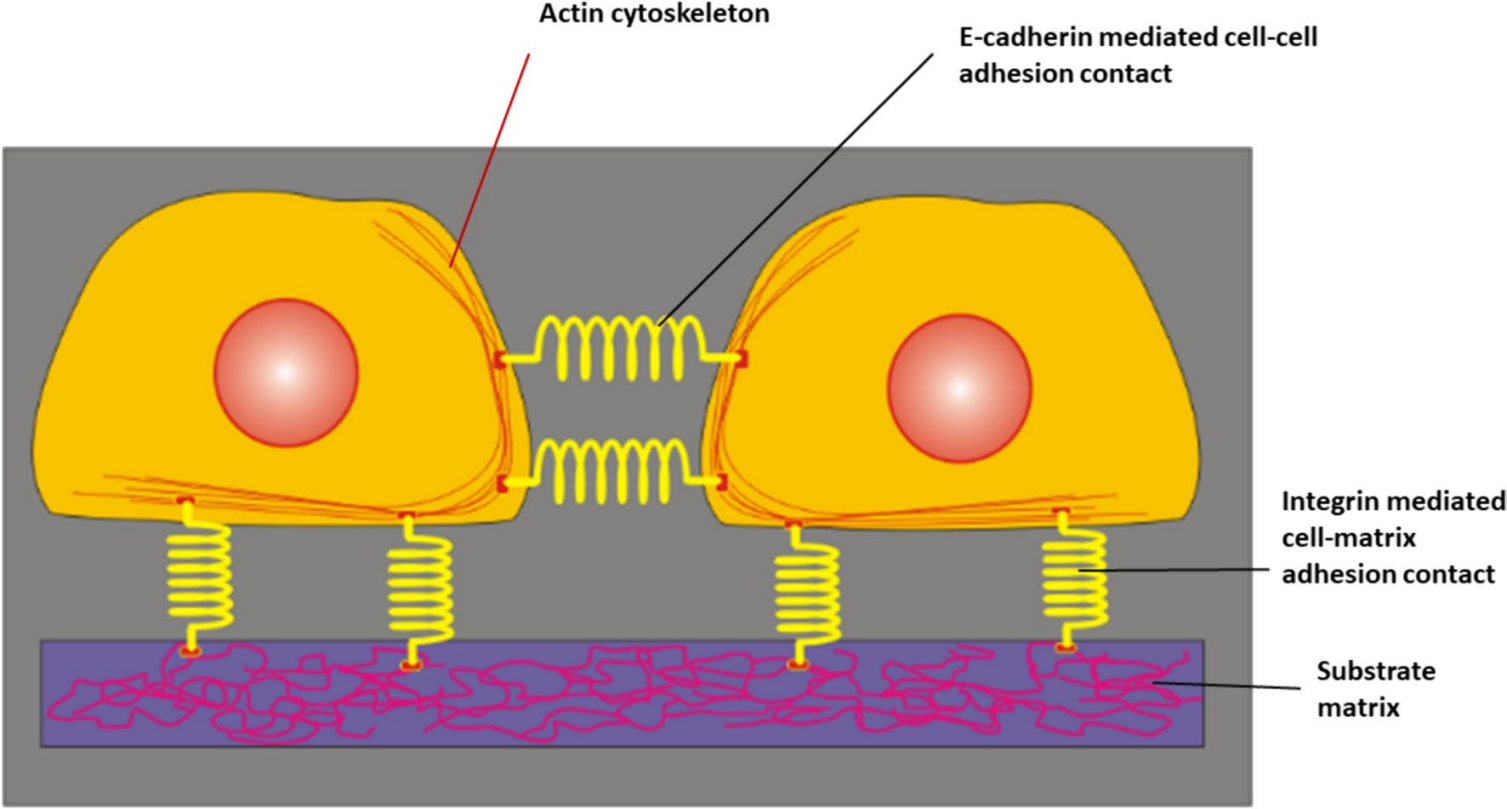
Interconnections between cell focal adhesions, adherens junctions, mediated by the actin cytoskeleton

The crosstalk between FAs and AJs is mediated by the actin cytoskeleton as shown in Fig. 2:

The rearrangement of these interconnected cellular elements occurs on a time scale of minutes. To maintain an ordered process of cell rearrangement, it is crucial for these cellular components to: (1) minimize the free energy expended during cell movement and (2) fluctuate around their equilibrium states caused by cell contractions (Wang et al. 2023). Consequently, the structural rearrangement of these interconnected elements requires $$\frac{{dF_{T} }}{dt} \to 0$$ (where $${F}_{T}$$ is the free energy equal to $$F_{T} = F_{AJs} + F_{FAs} + F_{cyt}$$, *F*_AJS_ is the free energy of AJs equal to $$ F_{{AJs}}  = \sum\nolimits_{i}^{{N^{ * } \left( \tau  \right)}} {F_{{AJi}} }  $$, *F*_FAs_ is the free energy of FAs equal to $$ F_{{FAs}}  = \sum\nolimits_{i}^{{N^{{ *  * }} \left( \tau  \right)}} {F_{{FAi}} }  $$, and *F*_cyt_ is the free energy of the cytoskeleton, $${N}^{*}$$ and $${N}^{**}$$ are the numbers of AJs and FAs per single cell, which change over a relatively long time scale of hours). The change of Helmholtz free energy of a single AJ can be expressed as: $$\frac{{dF_{AJ} }}{dt} = \Delta V_{AJ} \frac{{dW_{AJ} }}{dt} - T\frac{{dS_{AJ} }}{dt} + \left( {\frac{{\partial F_{AJ} }}{{\partial N_{cad} }}} \right)_{T} \frac{{dN_{cad} }}{dt}$$ (where $$t$$ is a relatively short time scale of minutes, W_AJ_ is the strain energy density of the FA, *T* is temperature, ∆V_AJ_ is the volume of a single AJ, S_AJ_ is the internal entropy generated within an AJ as a consequence of the interaction with the cytoskeleton and conformational changes of cadherin molecules under cell mechanical stress). The strain energy density ∆V_AJ_ accounts for the strain in cadherin–cadherin bonds and conformational changes of cadherin molecules and can be expressed as: $$W_{AJ} \left( {r_{ij} ,\theta_{ij} } \right) = \tilde{\sigma }_{AJ} :\tilde{\varepsilon }_{AJ}$$, (where θ_ij_ is the orientational angle between two cells, $$\tilde{\sigma }_{AJ}$$ is the intercellular stress on AJ, and $$\tilde{\varepsilon }_{AJ} = \tilde{\varepsilon }_{AJ} \left( {r_{ij} ,\theta_{ij} } \right)$$ is the extensional, compressional, or shear strain of an AJ. The strain energy density of an AJ depends on the average length of an intercellular bond, *r*_*ij*_ and the orientational angle between two cells *θ*_*ij*_. Change of the angle *θ*_*ij*_ is induced by generation of intercellular shear stress, while change of the length *r*_*ij*_ causes extension/compression of an AJ.

The change in Helmholtz free energy of a single FA can be expressed as: $\frac{{dF_{FA} }}{dt} = \Delta V_{FA} \frac{{dW_{FA} }}{dt} - T\frac{{dS_{FA} }}{dt} + \left( {\frac{{\partial F_{FA} }}{{\partial N_{cad} }}} \right)_{T} \frac{{dN_{cad} }}{dt}$ (where *W*_*FA*_ is the strain energy density of the FA, ∆V_FA_ is the volume of a single FA, *S*_*FA*_ is the internal entropy generated within the FA as a consequence of conformational changes of integrin and interactions with the cytoskeleton and substrate matrix under cell mechanical stress). The strain energy density *W*_*FA*_ accounts for the strain of integrin bonds established between ligands on the matrix surface, as well as for conformational changes of integrin molecules and can be expressed as: $W_{FA} \left( {d_{i} ,\theta_{ij} } \right) = \tilde{\sigma }_{FA} :\tilde{\varepsilon }_{FA}$, (where $\tilde{\sigma }_{FA}$ is the cell–matrix stress on FA and $\tilde{\varepsilon }_{FA} = \tilde{\varepsilon }_{FA} \left( {d_{i} } \right)$ is the extensional, compressional, or shear strain of the FA). The strain energy density of the FA depends on the average length of the cell–matrix bond *d*_*i*_ and the orientational angle between two cells *θ*_*ij*_. Changes of the angle *θ*_*ij*_ generate torsional shear stress on the FA, which can disrupt it, while changes in the length *d*_*i*_ can extend or compress the FA.

The change in the average length of intercellular bonds, the average length of cell–matrix bonds, and the orientational angle between two cells, depends on cell–cell interactions. Given that these interactions are influenced by changes in the states of AJs and FAs, it becomes imperative to probe deeper into the nature of these interactions due to their potential to initiate different cell rearrangement scenarios, including the epithelial-to-mesenchymal transition, live cell extrusion, and the cell jamming state transition. It is therefore necessary to examine these interactions in detail.

## Main characteristics of cell–cell interactions discussed on a cellular level

**Fig. 3:**
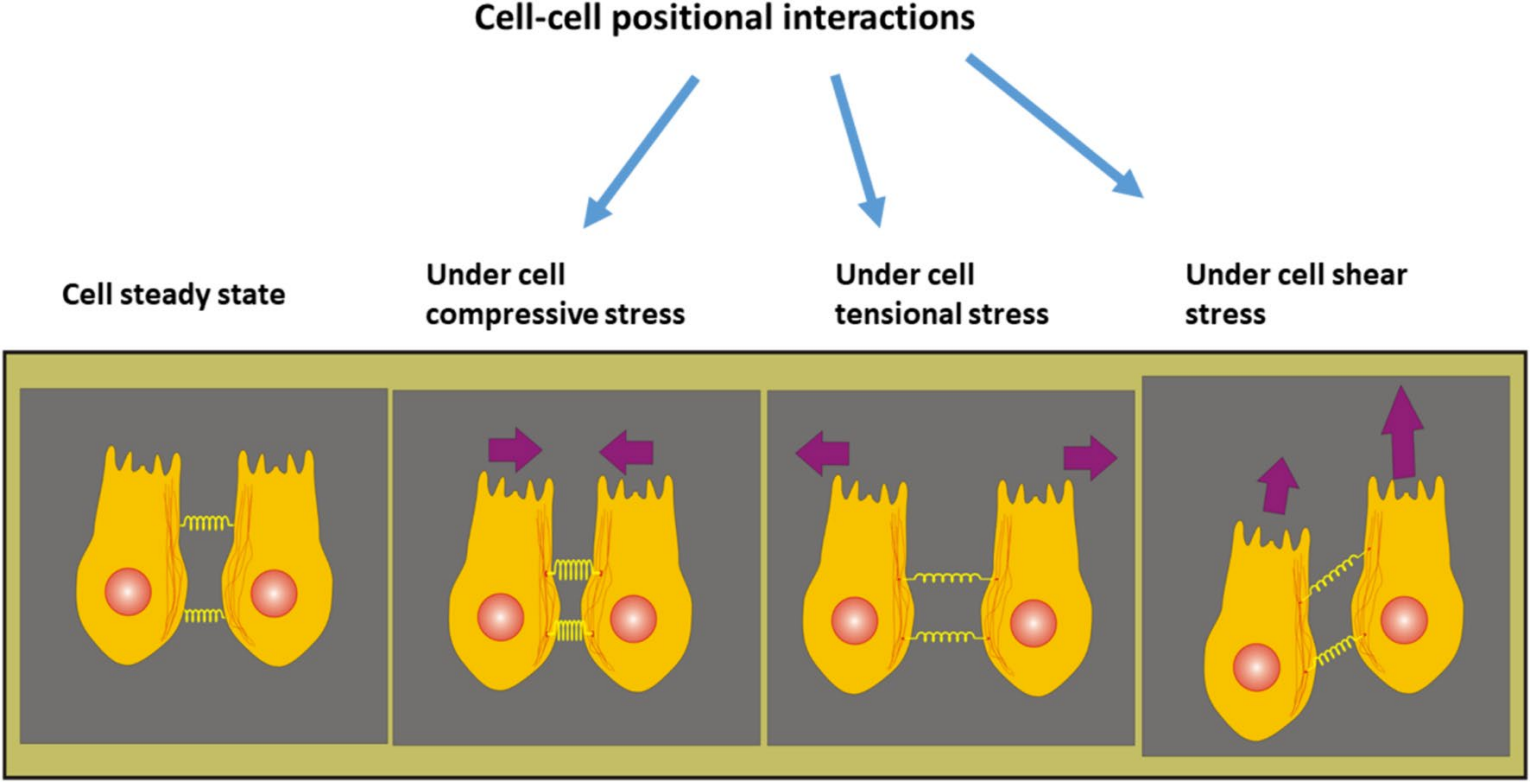
Schematic representation of the impact of cell mechanical stress on cell–cell positional interactions

**Fig. 4:**
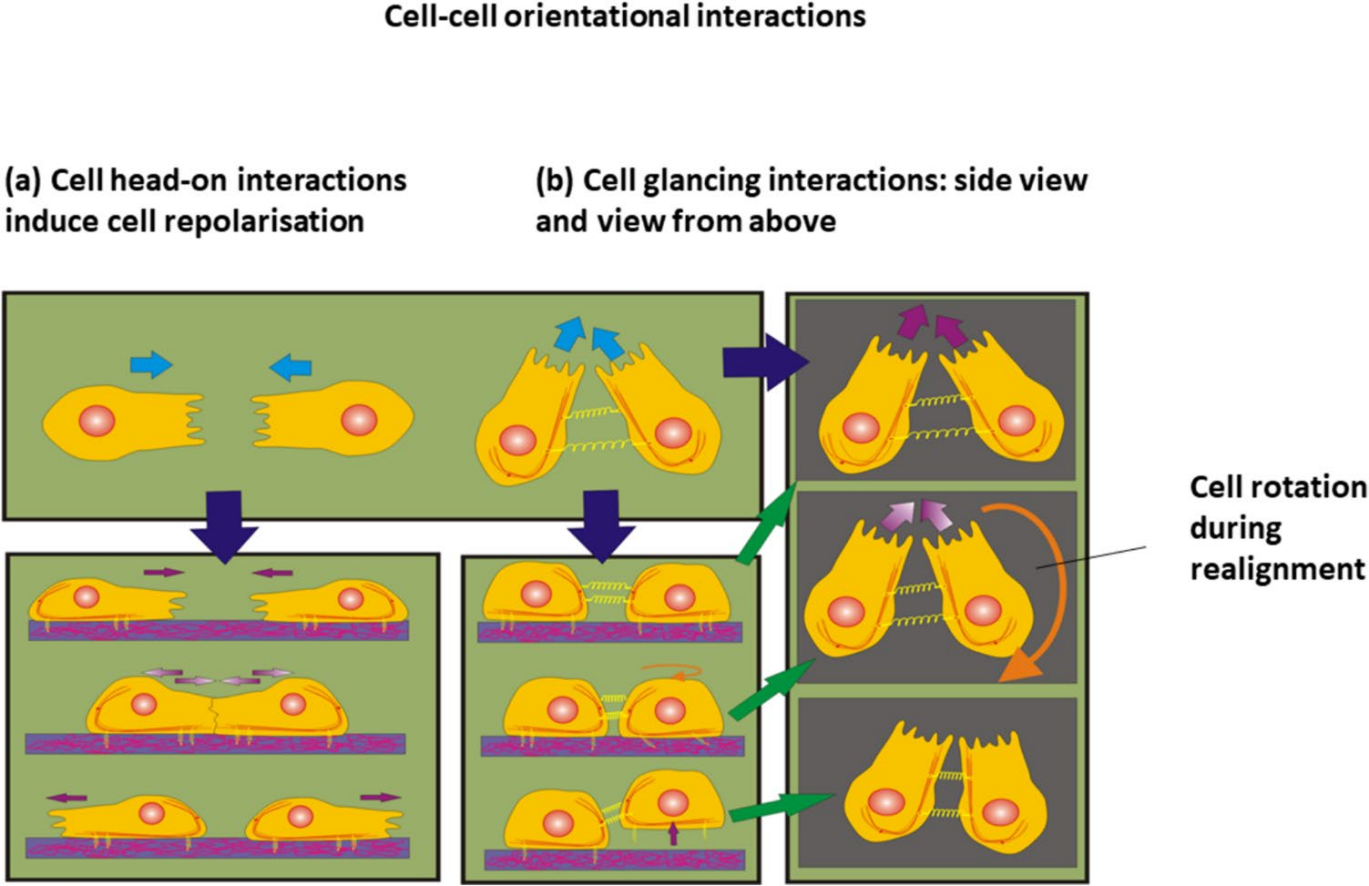
Schematic representation of cell–cell orientational interactions: **a** cell head-on interactions and **b** cell glancing interactions

Two types of cell–cell interaction have been distinguished during collective cell migration: positional and orientational, which are inter-dependent (Lin et al. 2018; Alert and Trepat 2020). Cell–cell positional interactions depend on the stretching/compression of cell–cell E-cadherin mediated adhesion contacts. Consequently, the interaction energy can be repulsive, when the local distance *r*_*ij*_ is shorter than the minimum distance *r*_*min*_. Deforet et al. (2014) proposed that $r_{\min } \approx 8 \mu m$. Cell positional interactions induces change in cell position and velocity caused by the accumulated cell mechanical stress, as shown in Fig. 3:

Cell–cell orientational interactions can repolarise the cell. This process depends primarily on the collision angle $\theta_{ij} = \theta_{i} - \theta_{j}$ between the two cells, (where θ_i_ and θ_i_ are the angles between $i$ th and $j$ th cell orientations, respectively relative to the direction of collective cell migration) (Lin et al. 2018). Head-on collisions occur for the angle of $\theta_{ij} \to \pi \pm \delta_{\theta }$, while glancing collisions occur for the angle of $0 < \theta_{ij} < \pi - \delta_{\theta }$. (where ${\delta }_{\theta }$ is the small angle, i.e., $\delta_{\theta } < \frac{\pi }{2}$). Head-on interactions induce contact inhibition of locomotion (CIL) resulting in cell repolarisation and migration in the opposite direction (Alert and Trapet, 2020). Cell repolarisation weakens the cell–cell and cell–matrix adhesion contacts (Roycraft and Mayor, 2016). Cell glancing interactions are not strong enough to induce cell repolarisation but lead to single cell rotation or to cell swirling motion (Lin et al. 2018). Cells retain strong E-cadherin-mediated cell–cell adhesion contacts during glancing interactions. Both types of cell orientational interactions are shown in Fig. 4:

The interplay between the two types of cell–cell interaction plays a crucial role in influencing cell rearrangement, specially in an overcrowded environment. In order to gain a comprehensive understanding of this cause-and-effect relationship, it is necessary to consider the intricacies of both types of interaction, highlighting their interdependence.

### Cell–cell positional interactions

Cell positional interactions change both the cell’s position within the space and its velocity. These interactions are influenced by cell mechanical stress (Fig. 3). Although some studies (Smeets et al. 2016; Lin et al. 2018; Koride et al. 2018) disregarded inertia, Deforet et al. (2014) considered inertial effects in simulations of 2D cell rearrangement using the Langevin equation. Long-term inertial effects have been discussed in the context of mechanical wave generation during collective cell migration (Notbohm et al 2016; Pajic-Lijakovic et al. 2024). Nevertheless, none of these studies addressed the viscoelastic properties of multicellular systems. At the cellular level, viscoelastic behavior can be incorporated through the generalized Langevin equation (Pajic-Lijakovic and Milivojevic 2022b). Although this equation has been utilized to explain mesoscopic dynamics in nonequilibrium multi-body thermodynamic systems (Budini and Caceres 2004; Meyer et al., 2021), we propose its application in modeling single-cell migration patterns. Cells exhibit a higher level of complexity compared to colloidal particle ensembles, with their responses to mechanical and biochemical cues involving a complex interplay between cell signaling and gene expression (Blanchard et al. 2019; Barriga and Mayor 2019).

Both processes operate within the same time frame as cell migration (Petrungaro et al. 2019). The expression of genes introduces a time delay in the cellular response (Petrungaro et al. 2019). The relevance of this delay is that it allows a cell to have acquired information from surrounding cells at a previous time point (Blanchard et al. 2019; Petrungaro et al. 2019). These perturbations can induce two outcomes: (1) cells within the same population responding to different signals, and/or (2) cells exhibiting distinct behaviors in response to the same signals (Blanchard et al. 2019). These perturbations may therefore lead to uncorrelated cell movements.

Consequently, single-cell movement requires incorporation of the delay time distribution in the form of the generalized Langevin equation (Pajic-Lijakovic and Milivojevic 2022b) as:

1 $$  m_{c} \frac{{d\vec{v}_{{ei}} \left( \tau  \right)}}{{d\tau }} =  - \int\limits_{0}^{\tau } {\xi \left( {\tau  - \tau ^{\prime}} \right)\vec{v}_{{ei}} \left( {\tau ^{\prime}} \right)d\tau ^{\prime} - \frac{{\partial U_{{\text{int} }} }}{{\partial \vec{r}_{i} }} + \vec{F}_{{di}} }  $$

where $\tau$ is a long-time of hours, $\vec{v}_{ei} \left( \tau \right) = \frac{{d\vec{r}_{i} }}{d\tau }$ is the velocity of the *i*th cell, $\vec{r}_{i}$ is the position of the *i*th cell, ${m}_{c}$ is the mass of a single-cell, ${U}_{int}$ is the cell–cell interaction potential, $\vec{F}_{di} = \vec{F}_{Pi} + \vec{F}_{RANi}$ is the stochastic driving force for single-cell movement $\vec{F}_{Pi}$, is the cell self-propulsion force expressed as $\vec{F}_{Pi} = F_{m} \vec{Q}_{i}$, $\vec{Q}_{i}$ is the single cell orientational vector expressed as $\vec{Q}_{i} = \cos \theta_{i} \overrightarrow { e}_{x} + \sin \theta_{i} \overrightarrow { e}_{y}$ (where $\vec{e}_{x}$ and $\vec{e}_{y}$ are unit vectors in x- and y-directions) (Smeets et al. 2016; Lin et al. 2018), F_m_ is the magnitude of the self-propulsion force (Smeets et al. 2016), $\vec{F}_{RANi}$ is the noise term, $\xi \left( \tau \right)$ is the memory kernel representing a delay time distribution (Budini and Caceres 2004). The latter is a consequence of the mechanical energy dissipation caused by cell–matrix and cell–cell interactions. The memory kernel $\xi \left( \tau \right) = \frac{{\left\langle {\vec{F}_{di} \left( {\tau + \Delta \tau } \right) \vec{F}_{di} \left( {\Delta \tau } \right)} \right\rangle }}{{\xi_{0} k_{B} T_{eff} }}$, ${\xi }_{0}$ is the friction coefficient, ${k}_{B}$ is Boltzmann’s constant, and ${T}_{eff}$ is the effective temperature (Budini and Caceras, 2004) which depends on the average cell speed, i.e. $\left( {k_{B} T_{eff} } \right)^{1/2} \sim \left\langle {\left\| {\vec{v}_{e} } \right\|} \right\rangle$ (Pajic-Lijakovic and Milivojevic 2021b).

The cell–cell positional interactions depend on stretching/compression of cell–cell E-cadherin mediated adhesion contacts, described by the interaction potential ${U}_{int}$, which accounts for attractive and repulsive interactions and has been described by the Lennard–Jones potential (Kang et al., 2021): ${U}_{int}\left({r}_{ij}\right)={k}_{int}\left[{\left(\frac{{r}_{min}}{{r}_{ij}}\right)}^{p}-{\left(\frac{{r}_{min}}{{r}_{ij}}\right)}^{q}\right]$ (where ${k}_{int}$ is the measure of cell–cell repulsive/attractive interactions, the average length intercellular bond length is ${r}_{ij}=\left|{\overrightarrow{r}}_{i}-{\overrightarrow{r}}_{j}\right|$, $p$ and $q$ are exponents, and ${r}_{min}$ is the characteristic cell–cell distance). Consequently, the interaction energy can be repulsive, when the local distance r is lower than the minimum distance ${r}_{min}$. The proposed values of the exponents are $p=6$ and $q=3$. The average length of intercellular bond can be stretched or compressed by cell mechanical stress caused by collective cell migration.

The cell propulsion force, which influences the cell–cell positional interactions, depends on the cell orientational vector.

### Cell–cell orientational interactions

Cell orientational interactions are caused by an interplay between the cell compressive and shear stress components. Although the compressive stress intensifies cell–cell collisions, shear stress perturbs cell alignment. The two types of cell orientational interaction, depending on the intercellular angle, i.e. cell glancing interactions and cell head on interactions (Fig. 4**)** will now be considered separately.

#### Cell–cell glancing interactions

Cell glancing interactions cause single cell rotation, which changes the angle between the cell and the direction of collective cell migration. The altered alignment of the single cell has been described by the linear form of the Langevin equation (Farrell et al. 2012; Smeets et al. 2016):

2 $$\frac{{d\theta_{i} \left( \tau \right)}}{d\tau } = - k_{\theta } \sum\limits_{j = 1}^{{N_{n} }} {F\left( {\theta_{ij} ,r_{ij} } \right) + \Gamma \left( \tau \right)}$$

where ${k}_{\theta }$ is the specific rate of angle change, which depends on the elasticity of E-cadherin mediated cell–cell adhesion contacts and is equal to ${k}_{\theta }=\frac{1}{{\tau }_{\theta }}$, ${\tau }_{\theta }$ is the relaxation time of cell realignment, ${N}_{n}$ is the number of neighbouring cells, $F\left({\theta }_{ij},{r}_{ij}\right)$ is the driving force for cell alignment, and $\Gamma \left( \tau \right)$ is the stochastic driving force, which depends on the magnitude of cell shear stress and is equal to: $\Gamma \left( \tau \right) = \sqrt {2D_{R} } \eta_{i}^{R} \left( \tau \right)$ (where ${D}_{R}$ is the rotation diffusion coefficient, which depends on the cell shear stress, i.e., ${D}_{R}={D}_{R}\left({\widetilde{\sigma }}_{erS}\right)$, ${\eta }_{i}^{R}\left(\tau \right)$ is the white noise). Changing the angle ${\theta }_{ij}$ can induce stretching of the cell–matrix bond by increasing the average length ${d}_{i}$. For small angles ${\theta }_{ij}$, the force $F\left({\theta }_{ij},{r}_{ij}\right)$ can be simplified to $F\left({\theta }_{ij},{r}_{ij}\right)\sim {\theta }_{ij}$ (Farrell et al. 2012). The torsional potential responsible for cell rotation during realignment can be expressed as: ${U}_{\theta }\left(\theta ,{r}_{ij}\right)={\int }_{0}^{\theta }F\left({\theta }_{ij},{r}_{ij}\right)d{\theta }_{ij}$.

The orientation vector ${\overrightarrow{Q}}_{i}\left({\theta }_{i}\right)$ can be averaged on some multicellular domain $r$: $\langle \overrightarrow{Q}\rangle \left(r,\tau \right)=\int {\overrightarrow{Q}}_{i} {\rho }_{\theta }d\theta$ (where ${\rho }_{\theta }$ is the cell angle distribution). The ensemble-averaged orientation vector $\left\langle {\vec{Q}} \right\rangle$ is associated with the external field Ѱ responsible for the cell orientation as:

3 $$\left\langle {\vec{Q}} \right\rangle \left( {r,\tau } \right) = \chi_{c} \vec{\Psi }$$

where ${\upchi }_{c}$ is a tissue susceptibility (Zemel and Safran 2007) and $\vec{\Psi }$ is the external conservative field (homogeneous within a given multicellular domain) expressed as: $\vec{\Psi } = l_{p} \vec{\nabla }\varphi ,$ ${l}_{p}$ is the cell persistence length and $\varphi$ is the dimensionless scalar field, which can be expressed as: (1) $\varphi \equiv \frac{{c\left( {r,\tau } \right)}}{\left\langle c \right\rangle }$ for chemotaxis (where $c\left(r,\tau \right)$ is local concentration of nutrients, $\left\langle c \right\rangle$ is the average concentration of nutrients, (2) $\varphi \equiv \frac{{E_{m} \left( {r,\tau } \right)}}{{\left\langle {E_{m} } \right\rangle }}$ for durotaxis (where ${E}_{m}\left(r,\tau \right)$ is the local Young’s modulus of the matrix and $\left\langle {E_{m} } \right\rangle$ is the averaged Young’s modulus), (3)$\varphi \equiv \frac{{S_{c} \left( {r,\tau } \right)}}{{\left\langle {S_{c} } \right\rangle }}$ for cell wetting/de-wetting where ${S}_{c}\left(r,\tau \right)$ is the cell spreading factor, which represents the difference between the adhesion and cohesion energies of epithelial monolayers, and (4) $\varphi \equiv \frac{{\rho_{m} \left( {r,\tau } \right)}}{{\left\langle {\rho_{m} } \right\rangle }}$ for haptotaxis (where ${\rho }_{m}\left(r,\tau \right)$ is the local surface density of cell–matrix adhesion contacts, i.e. focal adhesions and $\left\langle {\rho_{m} } \right\rangle$ is the average surface density of FAs), and many others (Murray et al. 1988; Shellard and Mayor 2020, 2021). In real situations, the directional cell movement is induced by an interplay between several scalar fields. Besides cell glancing interactions, head-on interactions also occur within migrating epithelial collectives.

#### Cell–cell head-on interactions

A head-on collision between two cells stimulates a signaling cascade resulting in cell repolarisation (Roycraft and Mayor, 2016). Cell repolarisation is associated with a series of biochemical processes leading to molecular transport phenomena that play a crucial role in the rearrangement of the cytoskeleton and the remodelling of adhesion contacts between cells and the extracellular matrix. These structural alterations at the subcellular level can be defined by variations in the internal energy of the cell. This observation aligns with the understanding that cells function as complex thermodynamically open systems, which are characterized by specific state variables (Lucia 2015). This type of cell–cell interactions has been experimentally verified in epithelial monolayers when forwards and backwards flows collide due to the inhomogeneous nature of collective cell migration (Serra-Picamal et al 2012; Pajic-Lijakovic and Milivojevic 2022a). The cell repolarisation can be treated as a phase transition with a constant angle ${\theta }_{ij}\to \pi \pm {\delta }_{\theta }$ between the two cells. Eyring transient state theory can be applied to describe cell repolarisation within a dense epithelial collective in the form:

4 $$\frac{d{\rho }_{Ei}\left(\tau \right)}{d\tau }=-{\uplambda }^{*}d{\rho }_{E0i}$$

where ${\rho }_{Ei}$ is the perturbed intrinsic energy density of an active contractile cell, ${\rho }_{E0i}$ is the intrinsic energy density of a single active contractile cell before the head-on collision, $\tau$ is the long time = scale of a few hours, ${\uplambda }^{*}=\uplambda {e}^{- \frac{\Delta {E}_{T}-\Delta {E}_{s}}{{k}_{B}{T}_{eff}}}$, $\uplambda$ is the collision frequency which depends on the average cell speed per multicellular domain and the cell packing density, i.e. $\uplambda \sim \langle \Vert {\overrightarrow{{\varvec{v}}}}_{{\varvec{e}}}\Vert \rangle { n}_{e}^{1/3}$ (Pajic-Lijakovic and Milivojevic 2021b).

The energy barrier $\Delta {E}_{T}$ represents the free energy of cell polarisation and is equal to $\sim {10}^{-12} \text{Joules}$ (Zhong et al. 2014). A reduction of this energy barrier can be achieved through a signaling cascade initiated by a direct collision between cells, as proposed by Roycraft and Mayor (2016). The energy caused by signaling can be expressed as: $\Delta {E}_{s}={k}_{B}{T}_{eff}\Delta H$ (where $\Delta H$ is the Shannon information entropy, which has been already applied to cell signaling and was expressed as: $\Delta H=H\left(I\right)-H\left(I/R\right)$ (Rhee et al. 2012), $H\left(I\right)$ can be interpreted to be the overall uncertainty about the input $I$ and $H\left(I/R\right)$ is the residual uncertainty about the input *I* after the value of the response $R$ is known. The Shannon information entropy increases with the frequency of cell–cell collisions.

The intrinsic energy density ${\rho }_{Ei}$ relaxes towards the steady energy ${\rho }_{E0i}$ after the cell head-on collision, i.e., ${\rho }_{Ei}\to {\rho }_{E0i}$ during the relaxation time ${\tau }_{R}\sim 1/{\uplambda }^{*}$, which corresponds to several tens of minutes (Notbohm et al. 2016). When the relaxation time ${\tau }_{R}$ is significantly longer than the time between two collisions ${\tau }_{c}=1/\lambda$, i.e., ${\tau }_{R}\gg {\tau }_{c}$, which is satisfied for $\Delta E_{T} \gg \Delta E_{s}$, the cell cannot repolarise and it undergoes the cell-jamming state transition (i.e., contractile to noncontractile cell state transition) (Pajic-Lijakovic and Milivojevic 2022a).

In contrast to cell head-on collisions, which trigger cell repolarisation under constant angle between two cells, cell glancing interactions induce cell rotation during its realignment resulting in a decrease in the angle ${\theta }_{ij}$. While cell repolarisation induces a weakening of cell–cell adhesion contacts, cells retain their cell–cell adhesion contacts during glancing collisions (Alert and Trepat 2020).

Cell–cell interactions have an impact on cellular configurations and velocities, which will be discussed in the form of temporal evolution of the probability density distribution $f\left(\overrightarrow{r},\tau ,{\overrightarrow{v}}_{e}\right)$. In this context, the number of cells $d{N}_{c}$ located between $\overrightarrow{{\varvec{r}}}$ and $\overrightarrow{{\varvec{r}}}+d\overrightarrow{{\varvec{r}}}$ with cell velocity between ${\overrightarrow{v}}_{e}$ and ${\overrightarrow{v}}_{e}+d{\overrightarrow{v}}_{e}$ can be expressed as: $d{N}_{c}=f\left(\overrightarrow{r},\tau ,{\overrightarrow{v}}_{e}\right){d}^{2}\overrightarrow{r}{d}^{2}{\overrightarrow{v}}_{e}$ (where ${d}^{2}\overrightarrow{r}{d}^{2}{\overrightarrow{v}}_{e}$ is the part of 2D phase space equal to ${d}^{3}\overrightarrow{r}{d}^{3}{\overrightarrow{v}}_{e}=dxdyd{v}_{ex}d{v}_{ey}$). The local cell packing density is equal to ${n}_{e}\left(r,\tau \right)=\frac{1}{{l}_{e}}{\int }_{\Delta A}f\left(\overrightarrow{r},\tau ,{\overrightarrow{v}}_{e}\right){d}^{2}{\overrightarrow{v}}_{e}$ (where ${l}_{e}$ is the average size of a single epithelial cell and $\Delta A$ is the multicellular surface domain), while the cell velocity at the mesoscopic level is equal to ${\overrightarrow{v}}_{e}\left(r,\tau \right)=\frac{1}{\Delta A}{\int }_{\Delta A}{\overrightarrow{v}}_{e }f\left(\overrightarrow{r},\tau ,{\overrightarrow{v}}_{e}\right){ d}^{2}\overrightarrow{r}$. The mesoscopic cell velocity ${\overrightarrow{v}}_{e}\left(r,\tau \right)$ can be further formulated as the rate of change of the cell displacement field and expressed as: ${\overrightarrow{v}}_{e}\left(r,\tau \right)=\frac{d\overrightarrow{u}\left(r,\tau \right)}{d\tau }$ (where $\overrightarrow{u}\left(r,\tau \right)$ is the cell local displacement field caused by collective cell migration).

## Mesoscopic kinetic model of cell rearrangement: the impact of cell–cell interactions

Cell–cell interactions influence cellular configurations and velocities (Alert and Trepat 2020). This cause–consequence relationship can be described in the form of the probability density distribution $f\left(\overrightarrow{r},\tau ,{\overrightarrow{v}}_{e}\right)$. The evolution of density $f\left(\overrightarrow{r},\tau ,{\overrightarrow{v}}_{e}\right)$ in the phase space can be expressed in the form of the modified Boltzmann transport equation discussed by Eftimie (2018):

5 $$\frac{\partial f}{{\partial \tau }} + \vec{\nabla } \cdot \left( {f\vec{v}_{e} } \right) = \left( {\frac{\partial f}{{d\tau }}} \right)_{coll}$$

where ${\left(\frac{\partial f}{d\tau }\right)}_{coll}$ accounts for cell collisions and can be expressed as (Eftimie, 2018):

6 $${\left(\frac{\partial f}{d\tau }\right)}_{coll}=-\uplambda f\left(\overrightarrow{r},\tau ,{\overrightarrow{v}}_{e}\right)+\lambda {\int }_{V}T\left({\overrightarrow{v}}_{e},{\overrightarrow{v}}_{e}^{\prime}\right)f\left(\overrightarrow{r},\tau ,{\overrightarrow{v}}_{e}^{\prime}\right)d{\overrightarrow{v}}_{e}^{\prime}$$

where ${\lambda }^{-1}$ is the mean time between two collisions and $T\left({\overrightarrow{v}}_{e},{\overrightarrow{v}}_{e}^{\prime}\right)$ is the kernel which describes the probability of change in the cell velocity from ${\overrightarrow{v}}_{e}$ to ${\overrightarrow{v}}_{e}^{\prime}$ by accounting for various types of cell–cell interaction. The kernel $T\left({\overrightarrow{v}}_{e},{\overrightarrow{v}}_{e}^{\prime}\right)$ depends on the accumulated cell mechanical stress.

For the cell head-on collision, two values of cell velocity (for the cell that undergoes repolarisation) could be taken into accounted: (1) ${\overrightarrow{v}}_{e}={v}^{*}$ before collision and (2) ${\overrightarrow{v}}_{e}={-v}^{*}$ after collision. In this case, the kernel $T\left({\overrightarrow{v}}_{e},{\overrightarrow{v}}_{e}^{\prime}\right)$ can be expressed as: $T\left({\overrightarrow{v}}_{e},{\overrightarrow{v}}_{e}^{\prime}\right)=\delta \left({\overrightarrow{v}}_{e}-{\overrightarrow{v}}_{e}^{\prime}\right)$. However, within a migrating epithelial collective both types of cell–cell collision influence cell rearrangement and the kernel $T\left({\overrightarrow{v}}_{e},{\overrightarrow{v}}_{e}^{\prime}\right)$ can be nonlinear. For describing the distribution of velocities within Madin–Darby canine kidney (MDCK) epithelial monolayers, Lin et al. (2021) proposed a 2D $q$-Gaussian distribution with $q\approx 1.2$ as a measure of the nonlinearity. This type of distribution points to the presence of multiplicative noise caused by cell–cell collisions.

Given that that the cell mechanical stress, accumulated during collective migration of epithelial cells, is a key physical factor influencing cell–cell interactions and, on that basis, the strength of AJs and FAs, it is necessary to discuss the causes of the mechanical stress generation.

## Cell mechanical stress generation during collective migration of epithelial monolayers

As mentioned above, collective migration of epithelial monolayers on substrate matrices has been considered as active wetting (extension)/de-wetting (compression). Whether an epithelial monolayer undergoes active wetting or de-wetting depends on the interplay between specific adhesion and cohesion energies, which can be expressed in the form of a cell spreading factor as: ${S}_{c}\left(r,\tau \right)={\omega }_{a}-{\omega }_{c}$ (where ${\omega }_{a}$ is the cell–matrix adhesion energy and ${\omega }_{c}$ is the cell cohesion energy) (Pajic-Lijakovic and Milivojevic 2023b). The local cell–matrix adhesion energy can be written ${\omega }_{a}\left(r,\tau \right)={\rho }_{a}\langle {e}_{FA}\rangle$, where $\tau$ is the time scale of hours, $r$ is the local position within epithelial monolayer, and ${\rho }_{a}$ is the surface density of integrin-mediated cell–substrate adhesion contacts (Murray et al. 1988). The cell–cell cohesion energy can be expressed as: ${\omega }_{c}\left(r,\tau \right)=2{\gamma }_{e}$ (where the epithelial surface tension ${\gamma }_{e}=\frac{\partial {E}_{S}}{\partial A}$, ${E}_{S}$ is the surface energy of the epithelial monolayer, and $A$ is the monolayer surface area). The surface energy of the epithelial monolayer includes several contributions, i.e., the elastic and contractile contributions and the contribution of cell–cell adhesion energy per cell. Consequently, the surface energy of epithelial monolayers was expressed as (Koride et al., 2019): $E_{S}  = \sum\nolimits_{i} {\Delta A_{i}^{2} }  + \sum\nolimits_{{i,j}} {\Lambda r_{{ij}} }  + \sum\nolimits_{i} {e_{{conti}} }$ (where $K$ is the effective modulus of the cell around its preferred surface area, $\Delta {A}_{i}$ is the change in surface area per cell, ${r}_{ij}$ is the average length of an intercellular bond, $\Lambda$ is the line tension per unit interface length between two cells equal to $\Lambda = \left\langle {\rho_{eL,} e_{AJ} } \right\rangle$, ${\rho }_{eL}$ is the line density E-cadherin mediated adhesion contacts between two neighbouring cells, and ${e}_{cont}$ is the cell contractile energy. When ${\omega }_{a}\left(r,\tau \right)>{\omega }_{c}\left(r,\tau \right),$ cells undergo wetting, while de-wetting occurs when ${\omega }_{a}\left(r,\tau \right)<{\omega }_{c}\left(r,\tau \right)$. Inhomogeneous epithelial wetting/de-wetting is the main generator of cell mechanical stress depending on the viscoelasticity and surface characteristics of both cell monolayer and substrate matrix expressed in the form of the epithelial–matrix interfacial tension (Pajic-Lijakovic and Milivojevic 2023b; Pajic-Lijakovic et al. 2024).

### Cell mechanical stress: modeling consideration

The viscoelasticity of epithelial monolayers, and the interfacial tension between cells and the extracellular matrix, play crucial roles in determining the mechanical stress generated during collective cell migration (Pajic-Lijakovic and Milivojevic 2020). The strength of cell–cell and cell–matrix adhesion contacts, cell contractility, and stiffness of the substrate matrix are key factors that influence both, the viscoelasticity of epithelial monolayers and cell–matrix interfacial tension (Pajic-Lijakovic and Milivojevic 2023b). The cell–matrix interfacial tension is influenced by the epithelial surface tension, matrix surface tension, and cell–matrix adhesion energy. This physical parameter varies over time and can be defined as:

7 $${\gamma }_{em}\left(r,\tau \right)={\gamma }_{e}\left(r,\tau \right)+{\gamma }_{m}\left(r,\tau \right)-{\omega }_{a}\left(r,\tau \right)$$

where ${\gamma }_{m}\left(r,\tau \right)$ is the matrix surface tension and ${\gamma }_{e}\left(r,\tau \right)=\frac{1}{2}{\omega }_{c}$ is the epithelial surface tension. The epithelial surface tension depends on the strength of cell–cell adhesion contacts and cell contractility (Devanny et al. 2021). Extension of E-cadherin-mediated cell–cell adhesion contacts enhances the epithelial surface tension (Devanny et al. 2021). The equilibrium (static) tissue surface tension obtained after uni-axial compression of cell aggregates is in the range from a few $\frac{mN}{m}$ to several tens of $\frac{mN}{m}$ (Mombash et al., 2005; Stirbat et al. 2013; Efremov et al. 2021). The epithelial surface tension changes within the monolayer due to the inhomogeneous distribution of cell–cell adhesion contacts (Pérez-González et al. 2019). The surface tension of the matrix such as collagen I depends on the concentration of collagen fibers. A decrease in concentration from $4\frac{mg}{{ml}}$ to $1\frac{mg}{{ml}}$ causes an increase in the static surface tension of collagen I matrix from $57\frac{mN}{m}$ to $62\frac{mN}{m}$ at 21 °C (for experiments conducted in the absence of cells) (Kezwon and Wojciechowski 2014). Tractions of epithelial monolayers on collagen I matrix, during collective cell migration, induce inhomogeneous distributions of collagen fibers and the establishment of a matrix surface tension gradient (Clark et al. 2022; Pajic-Lijakovic, 2023b). Consequently, inhomogeneous distributions of the epithelial surface tension, matrix surface tension, and cell–matrix adhesion energy along migrating cell monolayers, generate a cell–matrix interfacial tension gradient $\vec{\nabla }\gamma_{em}$ and its change on a time-scale of hours. Both parameters, the interfacial tension and the gradient of interfacial tension, accompanied by the viscoelasticity caused by collective cell migration influence the generation of cell residual mechanical stress (Pajic-Lijakovic and Milivojevic 2023b). Its accumulation within monolayers includes both normal (tensional/compressive) and shear components (Serra-Picamal et al. 2012; Tambe et al., 2013; Notbohm et al. 2016).

The cell residual stress consists of two main components: isotropic and deviatoric stress. The isotropic stress is induced by the cell–matrix interfacial tension, as described by the Young–Laplace equation. On the other hand, the deviatoric stress results from coordinated cell movement. Therefore, the overall cell normal residual stress can be represented as (Pajic-Lijakovic and Milivojevic 2023b):

8 $$\tilde{\sigma }_{erV} = \pm \Delta p_{e \to m} \tilde{I} + \tilde{\sigma }_{erV}^{CCM}$$

**Fig. 5:**
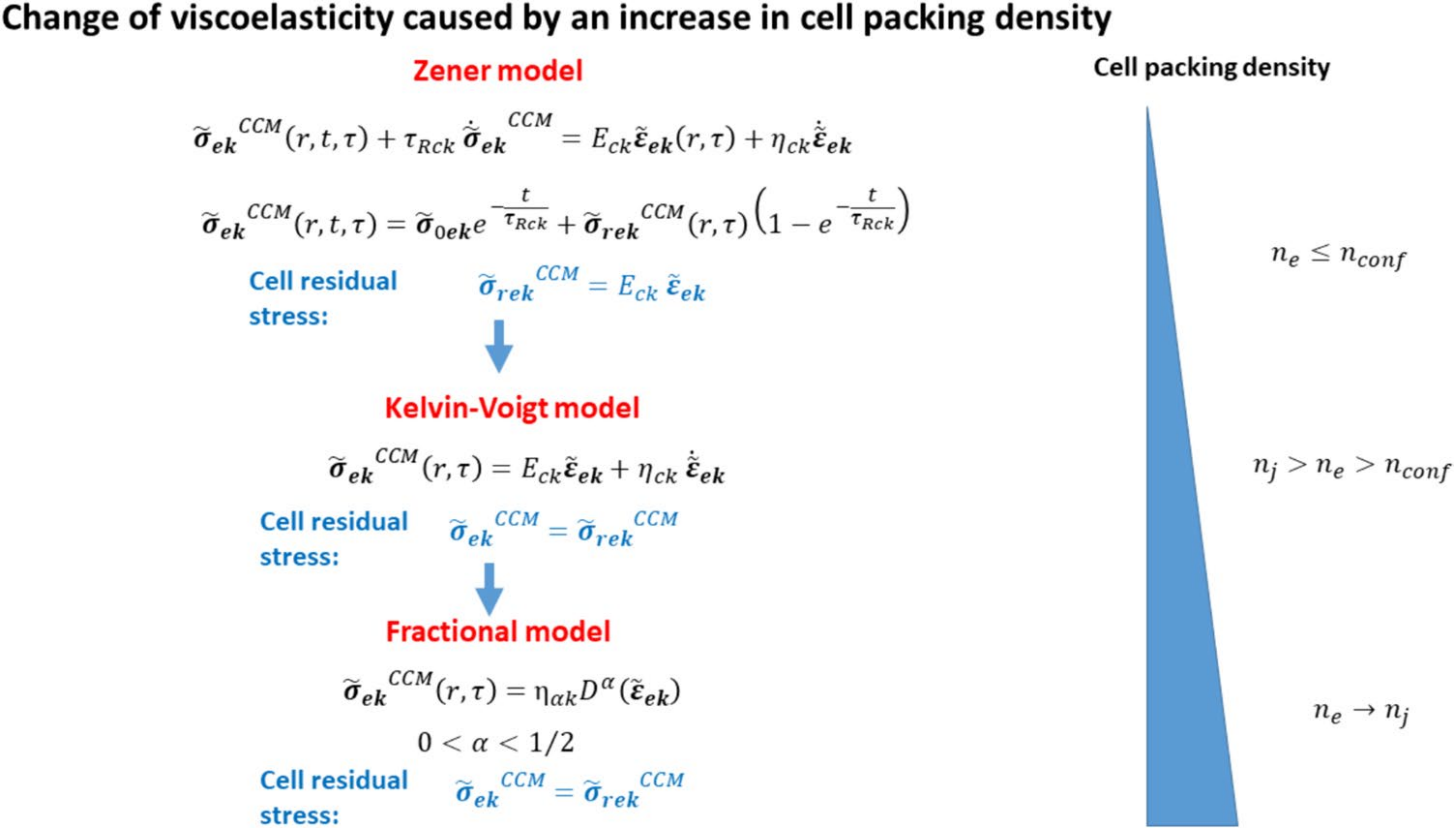
Viscoelasticity caused by collective cell migration: constitutive models depending on the cell packing density, where ${n}_{conf}$ is the cell packing density in the confluent state, ${n}_{j}$ is the cell packing density in the jamming state, the subscript $k\equiv S,V$, such that $S$ is shear stress/strain component, $V$ is volumetric stress/strain component, ${\tau }_{Rck}$ is the cell stress relaxation time, ${E}_{ck}$ is the elastic modulus, ${\upeta }_{ck}$ is the cell viscosity (shear or bulk), $r$ is the space coordinate, $t$ is a short-time scale (i.e. minutes), $\tau$ is a long-time-scale (i.e. hours), $\overrightarrow{u}\left(r,\tau \right)$ is the cell local displacement field, ${{\widetilde{\sigma }}_{ek}}^{CCM}\left(r,t,\tau \right)$ is the cell stress (normal or shear) caused by collective cell migration, ${\widetilde{\sigma }}_{0ek}$ is the initial value of the cell stress, ${{\widetilde{\sigma }}_{ek}}^{CCM}$ is the cell residual stress part caused by collective cell migration, ${{\dot{\widetilde{\sigma }}}_{ek}}^{CCM}$ is the rate of stress change ${{\dot{\widetilde{\sigma }}}_{ek}}^{CCM}=\frac{d{{\widetilde{\sigma }}_{ek}}^{CCM}}{dt}$ caused by the stress relaxation, ${\widetilde{\varepsilon }}_{ck}$ is the cell strain such that the volumetric strain is equal to ${\widetilde{\varepsilon }}_{eV}\left(r,\tau \right)=\overrightarrow{(\nabla }\bullet \overrightarrow{u})\widetilde{I}$, $\widetilde{I}$ is the unit tensor, the shear strain ${\widetilde{\varepsilon }}_{eS}\left(r,\tau \right)=\frac{1}{2}\left(\overrightarrow{\nabla }\overrightarrow{u}+{\overrightarrow{\nabla }\overrightarrow{u}}^{T}\right)$, ${\dot{\widetilde{\varepsilon }}}_{ck}$ is the corresponding strain rate equal to ${\dot{\widetilde{\varepsilon }}}_{ek}=\frac{d{\widetilde{\varepsilon }}_{ek}}{d\tau }$, ${\upeta }_{\alpha k}$ is the effective modulus, ${D}^{\alpha } \widetilde{\varepsilon }\left(r,\tau \right)=\frac{{d}^{\alpha }\widetilde{\varepsilon }\left(r,\tau \right)}{d{\tau }^{\alpha }}$ is the fractional derivative, and α gives the order of fractional derivatives (the damping coefficient). Caputo’s definition of the fractional derivative of a function $\widetilde{\varepsilon }\left(r,\tau \right)$ is used and expressed as: $D^{\alpha } \tilde{\varepsilon } = \frac{1}{{\Gamma \left( {1 - \alpha } \right)}}\frac{d}{dt}\mathop \smallint \limits_{0}^{t} \frac{{\tilde{\varepsilon }\left( {r,\tau^{\prime}} \right)}}{{\left( {\tau - \tau^{\prime}} \right)^{\alpha } }}d\tau^{\prime}$ (where Г $\left(1-\alpha \right)$ is a gamma function) (Podlubny, 1999)

Where $\tilde{\sigma }_{erV}$ is the cell normal residual stress component, $\tilde{I}$ is the unity tensor, $\Delta p_{e \to m}$ is the isotropic part of the cell normal stress equal to $\Delta p_{e \to m} = - \gamma_{em} \left( {\vec{\nabla } \cdot \vec{n}} \right)$, $\overrightarrow{n}$ is the normal vector of the cell–matrix biointerface, and $\tilde{\sigma }_{erV}^{CCM}$ is the deviatoric part of the cell normal residual stress, presented in Fig. 5. The tension and compression in the isotropic stress part are indicated by the positive and negative signs, respectively. The deviatoric part of the normal stress is influenced by the viscoelasticity of epithelial monolayers. Moreover, the viscoelasticity is dependent on the cell packing density and the strength of the cell–cell adhesion contacts, which will be thoroughly discussed below. The heterogeneous distribution of cell normal stress, generated during collective cell migration, results in an inhomogeneous distribution of cell packing density within monolayers (Nnetu et al. 2013; Pajic-Lijakovic and Milivojevic 2021a). While cell compressive stress causes an increase in the cell packing density, cell tensional stress induces a decrease in the cell packing density. An increase in cell packing density intensifies positional and orientational cell–cell interactions, accompanied by the contact inhibition of locomotion.

The cell shear residual stress is composed of two distinct parts. One is a consequence of natural convection resulting from the gradient in interfacial tension $\vec{\nabla }\gamma_{em}$, while the other part is due to forced convection, specifically collective cell migration. The extension of cells, either actively or passively, from regions of lower interfacial tension to regions of higher interfacial tension within multicellular domains is associated with the Marangoni effect (Pajic-Lijakovic and Milivojevic 2022c). Experimental validation of cell movement along multicellular surfaces induced by the gradient in surface tension has been demonstrated by Gsell et al. (2023). The Marangoni effect has been identified in various soft matter systems exposed to temperature or concentration gradients (Karbalaei et al., 2016).

Therefore, the shear residual stress can be formulated as:

9 $$\vec{n} \cdot \tilde{\sigma }_{erS} \cdot \vec{t} = \vec{\nabla }\gamma_{em} \cdot \vec{t} + \vec{n} \cdot \tilde{\sigma }_{erS}^{CCM } \cdot \vec{t}$$

Where $\tilde{\sigma }_{erS}$ is the cell shear residual stress component, $\tilde{\sigma }_{erS}^{CCM}$ is the cell shear stress generated by collective cell migration (Fig. 5), and $\vec{t}$ is the tangent vector of the cell–matrix biointerface. Cell shear stress intensifies glancing interactions, which can result in the disruption of cell–cell and cell–matrix adhesion contacts. Saw et al. (2017) pointed out that the generation of topological defects in cell alignment within cell monolayers, as a prerequisite of cell extrusion, is induced by cell compressive and shear stress components. Shear stress is also the one of the main factors responsible for the epithelial-to-mesenchymal transition (Rizvi et al., 2013; Pajic-Lijakovic and Milivojevic 2022b).

The magnitude of cell shear/normal residual stress resulting from collective cell migration is contingent upon the specific mechanism of cell migration, which in turn is determined by the cell packing density. Epithelial cell migration can be attributed to three mechanisms: (1) the convective mechanism for the cell packing density $n_{e} \le n_{conf}$, (2) the diffusion mechanism for the cell packing density $n_{conf} < n_{e} < n_{j}$, and (3) the sub-diffusion mechanism for the cell packing density $n_{c} \sim n_{j}$ (where *n*_*conf*_ is the cell packing density in the confluent state equal to ~2.5×10^5^ cells/cm^2^ (Petitjean et al. 2010) and ${n}_{j}$ is the cell packing density in the jamming state ~1×10^6^ cells/cm^2^ (Kaliman et al. 2021). The constitutive models for the various mechanisms of epithelial cell migration are illustrated in Fig. 5:

The reorganization of MDCK cell monolayers with a cell packing density $n_{e} \le n_{conf}$ was investigated by Serra-Picamal et al. (2012) and Notbohm et al. (2016). Their research revealed a direct correlation between the long-term cell stress, referred to as cell residual stress, and the corresponding strain. Accordingly, this finding suggests that these cells can be characterized as viscoelastic solids. The presence of strong cell–cell adhesion contacts mediated by E-cadherin in epithelial cells supports this observation. Another noteworthy characteristic of epithelial monolayers in this cell packing density range is their ability to relax stress towards the cell residual stress. Khalilgharibi et al. (2019) reported that stress relaxation occurs within minutes, while the accumulation of cell residual stress takes several hours. Marmottant et al. (2009) also observed stress relaxation in cell aggregates under uni-axial compression, providing further evidence for stress relaxation in these systems. Pajic-Lijakovic and Milivojevic (2020) concluded that changes in cell stress occur through successive short-time stress relaxation cycles, while the cell strain and corresponding cell residual stress change over hours. The Zener model, as presented in Fig. 5, could be a suitable constitutive model that satisfies the conditions of stress relaxation on a time scale of minutes and correlation between cell residual stress and strain, indicating long-term elastic behaviour (Pajic-Lijakovic and Milivojevic 2022a). Energy dissipation, characteristic of the viscoelastic behavior of multicellular systems, occurs over minutes due to the remodeling of cell–cell adhesion contacts (Pajic-Lijakovic and Milivojevic 2023a). The cell stress relaxes towards the elastic cell residual stress, while the cell residual stress, cell velocity, and strain oscillate over hours, as discussed in the context of mechanical waves (Serra-Picamal et al. 2012; Notbohm et al. 2016; Pajic-Lijakovic and Milivojevic 2020).

When the cell packing density increases further, within the range of $n_{conf} < n_{e} < n_{j}$, it results in the suppression of stress relaxation within the cells. Intensive friction between cells, which is a characteristic of higher cell packing densities, causes energy to dissipate over a prolonged period during the rearrangement of cells. Since cell movement is governed by a linear diffusion mechanism, the corresponding constitutive model used should also be linear. Pajic-Lijakovic and Milivojevic (2022a) proposed the Kelvin–Voigt constitutive model for this specific regime (Fig. 5). The corresponding long-term change in cell stress takes into account both the elastic and viscous contributions.

Cell movement in an overcrowded environment is damped and is governed by a sub-diffusion mechanism which has been described by fractional derivatives. Pajic-Lijakovic and Milivojevic (2021a) proposed the fractional constitutive model for describing the viscoelasticity of cell monolayers in this case (Fig. 5).

While the generated compressive stress intensifies cell–cell interactions, cell shear stress perturbs cell alignment, which generating topological defects in cell alignment (Saw et al. 2017).

## Topological defects of cell alignment within monolayers caused by cell mechanical stress

Cell glancing and head-on collisions are intensive within the disordered regions characterized as topological defects. These regions are generated by cell compressive and shear stress components and by mixing with surrounding ordered regions (Fig. 1). An increase in the surface fraction of the disordered regions can be described by the modified phase model B proposed for thermodynamic systems far from equilibrium (Tanaka et al., 1997; Ala Nissila et al., 2004). It is expressed as:

10 $$\frac{{\partial \phi_{e} \left( {r,\tau } \right)}}{\partial \tau } = \vec{\nabla }\left[ {D_{eff} \left( {\phi_{e} \vec{\nabla }\frac{{\delta F_{rear} \left( {\phi_{e} } \right)}}{{\delta \phi_{e} }}} \right) + \nabla \cdot \tilde{\sigma }_{RT} } \right]$$

where ${\upphi }_{e}$ is the surface fraction of disordered regions within epithelial monolayers, ${D}_{eff}$ is the effective dispersion coefficient, ${\widetilde{\sigma }}_{erT}={\widetilde{\sigma }}_{erS}+{\widetilde{\sigma }}_{erV}$ is the accumulated cell mechanical stress caused by collective cell migration, and $F\left({\phi }_{e}\right)$ is the free energy of the rearrangement of epithelial cells which, based on the modified model of Cohen and Murray (1981), can be expressed as:

11 $$  F_{{rear}} \left( {\phi _{e} } \right) = \int {\left[ {f_{{mix}} \left( {\phi _{e} } \right) + \frac{1}{2}k_{e} \left( {\vec{\nabla }\phi _{e} } \right)^{2} } \right]d^{3} r}  $$

Where $f_{mix} \left( {\phi_{e} } \right)$ represents the mixing energy contribution, while the second term describes effects along the biointerface between the ordered and disordered regions, and *k*_*e*_ represents a measure of cell–cell interactions along the biointerface between the regions caused by inhomogeneous wetting/de-wetting of epithelial monolayers. The free energy of mixing can be described by the modified Flory–Huggins relation, proposed for mixing of polymer blends, as $f_{mix} \left( {\phi_{e} } \right) = \frac{{k_{B} T_{eff} }}{{V_{e} }}\left[ {\phi_{e} \ln \phi_{e} + \left( {1 - \phi_{e} } \right) \ln \left( {1 - \phi_{e} } \right) + \chi \phi_{e} \left( {1 - \phi_{e} } \right)} \right]$, the single cell volume $V_{e} = \frac{1}{{n_{e} }}$, *χ* is the Flory–Huggins interaction parameter including both enthalpic and entropic contributions, i.e., $\chi = \chi_{S} + \chi_{H}$ where *χ*_*s*_ is the entropic contribution and *χ*_*H*_ is enthalpic contribution (Tambasco et al. 2006). For overcrowded cellular systems, the entropic contribution caused by cell–cell orientational interactions can be expressed as: $\chi_{S} = \frac{{\left\langle {U_{\theta } } \right\rangle^{r} }}{{\left\langle {U_{\theta } } \right\rangle^{T} }}$, $\text{where} {\langle {U}_{\theta }\rangle }^{r}$ is the averaged torsional potential per $r$ th domain and $\left\langle {U_{\theta } } \right\rangle^{T}$ is the averaged torsional potential per whole epithelial monolayer. The corresponding enthalpic contribution can be expressed as: $\chi_{H} = \frac{{U_{{\text{int}}} }}{{2k_{B} T_{eff} }}$.

The local internal entropy production during cell rearrangement can be expressed as:

12 $$\frac{{dS_{rear} \left( {r,\tau } \right)}}{d\tau } = \left( {\frac{{\partial S_{rear} }}{{\partial n_{e} }}} \right)_{{T_{eff} ,\left| {\vec{Q}} \right|}} \frac{{dn_{e} }}{d\tau } + \left( {\frac{{\partial S_{rear} }}{{\partial T_{eff} }}} \right)_{{n_{e} ,\left| {\vec{Q}} \right|}} \frac{{dT_{eff} }}{d\tau } + \left( {\frac{{\partial S_{rear} }}{{\partial \left| {\vec{Q}} \right|}}} \right)_{{T_{eff} ,n_{e} }} \frac{{d\left| {\vec{Q}} \right|}}{d\tau }$$

where ${S}_{rear}$ is the local entropy caused by cell rearrangement and $\frac{{dS_{rear} \left( {r,\tau } \right)}}{d\tau }$ is its rate of production. The production rate rises with increasing cell packing density${n}_{e}$, effective temperature $T_{eff} \sim \left\langle {\left\| {\vec{v}_{e} } \right\|} \right\rangle^{2}$ and orientational parameterr $\left| {\vec{Q}} \right|$. Noting (1) that the effective temperature and cell speed oscillate (Notbohm et al. 2016; Pajic-Lijakovic et al., 2020), (2) that changes in cell packing density are caused by an accumulation of cell compressive stress (Saw et al. 2017; Pajic-Lijakovic and Milivojevic 2023a), (3) that cell compressive stress oscillates with a long time period (Notbohm et al. 2016), and (4) that the orientational parameter fluctuates due to successive perturbations of cell alignment and realignment (Lin et al., 2016), it is evident that the internal production of entropy $\frac{{dS_{rear} \left( {r,\tau } \right)}}{d\tau }$ must also oscillate.. Change of cell packing density can be: (1) $\frac{{dn_{e} }}{d\tau } > 0$ for the extension of cell monolayer parts caused by wetting, (2) $\frac{{dn_{e} }}{d\tau } < 0$ for the compression of cell monolayer parts caused by de-wetting and $\frac{{dn_{e} }}{d\tau }\sim 0$ under cell shear stress, which has no impact on the cell packing density. An increase in the cell packing density caused by an accumulation of cell compressive stress, leads to a decrease in cell speed accompanied by the effective temperature. Consequently, when $\frac{{dn_{e} }}{d\tau } > 0$, than $\frac{{dT_{eff} }}{d\tau } < 0$. A decrease in the cell packing density, caused by an accumulation of cell tensional stress, leads to an increase in cell speed accompanied by the effective temperature. Consequently, when $\frac{{dn_{e} }}{d\tau } < 0$ than $\frac{{dT_{eff} }}{d\tau } > 0$. It is known that cell speed correlates with cell packing density in the form of $\left\| {\vec{v}_{e} } \right\|\sim n_{e}^{ - b}$ (where $b$ is the scaling exponent). The scaling exponent is: (1) b=1.85 for free expansion of MDCK cells (Tlili et al. 2018) and (2) b=2.35 for free expansion of MCF-10A cells (Nnetu et al. 2013). An increase in cell packing density can induce cell misalignment causing $\frac{{d\left| {\vec{Q}} \right|}}{d\tau } > 0$, while the cell realignment leads to $\frac{{d\left| {\vec{Q}} \right|}}{d\tau } < 0$. Cell–cell interactions, intensified by cell compressive and shear stress components, can perturb the state of cell–cell and cell matrix adhesion contacts, leading to various scenarios of cell rearrangement.

## Interrelationship between various scenarios of cell rearrangement and cell–cell interactions caused by an accumulation of cell mechanical stress

**Fig. 6:**
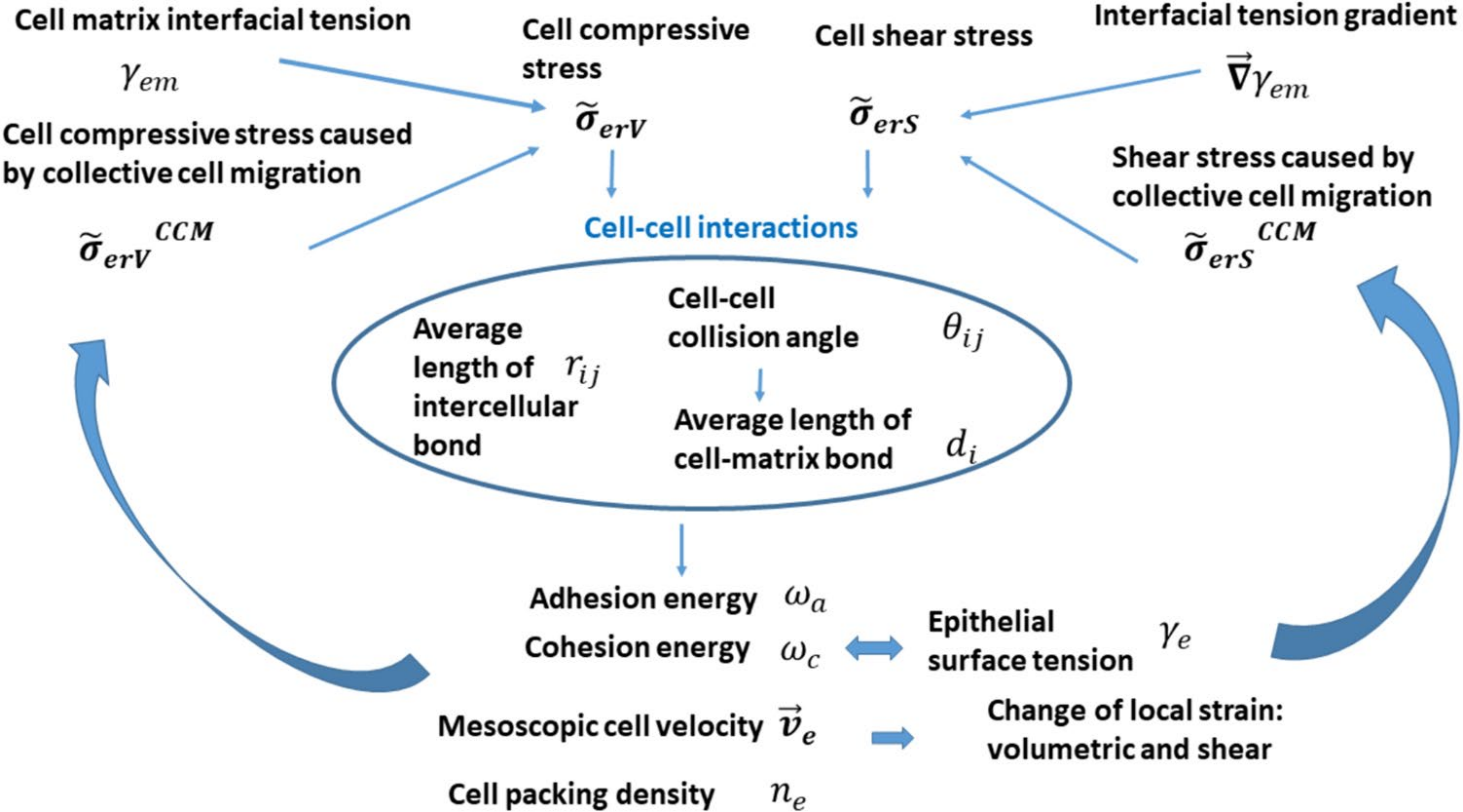
The interrelationships between: (1) the state of AJs and FAs and cell–cell interactions, and (2) cell–cell interactions and cell mechanical stress

Cell–cell interactions, generated by cell mechanical stress, influence the free energy of AJs and FAs by changing the average length of intercellular bonds ${r}_{ij}$, the average length of cell–matrix bonds ${d}_{i}$, and the intercellular collision angle ${\theta }_{ij}$. The interrelationships between: (1) the state of AJs and FAs and cell–cell interactions, and (2) cell–cell interactions and cell mechanical stress are shown schematically in Fig. 6:

An alteration in the distance ${r}_{ij}$ induces stretching/compression of intercellular E-cadherin mediated bonds, whereas a change in the angle ${\theta }_{ij}$ triggers a conformational change of cadherin molecules. These processes initiate an intercellular signalling cascade, which can boost phosphorylation of the cytoskeleton and the cadherin endocytosis resulting in a decrease in the number of cadherin molecules ${N}_{cad}$ per AJ, thereby weakening adherens junction (Barriga and Mayor 2019). Cell AJs are resistant to the tensional intercellular stress of $\sim 1 \text{kPa}$ (Lin et al. 2018). However, cell shear stress less than $1 \text{Pa}$ is enough to stimulate the epithelial-to-mesenchymal transition (Espina et al., 2023).

Change in the angle ${\theta }_{ij}$ is connected with the perturbation of cell alignment, caused by the interplay between cell shear and compressive stress components. If unaligned cells retain their AJs, change in angle ${\theta }_{ij}$ leads to an inhomogeneous stretching/compression of AJs. Some cadherin–cadherin bonds are more stretched than others, while some of them can be compressed at the same time, leading to the rotation of individual cells and ultimately causing cell realignment. Stretched semi flexible filaments such as cadherin molecules, exert larger forces than the compressed ones under the same absolute deformation (Broedersz and MacKintosh 2014). The cell rotation generates cell–matrix torsional shear stress, leading to stretching of an FA accompanied by an increase in the distance ${d}_{i}$. This torsional stress causes conformational changes of integrin molecules and can result in detachment of FAs, which can trigger the signalling cascade necessary for live cell extrusion. The detachment of an FA can be induced by an interfacial shear stress between the cell and the matrix, typically ranging from 4 to 6 Pa (Paddillaya et al., 2019).

Cell head-on interactions in an overcrowded environment cause a decrease in the cell–cell distance ${r}_{ij}$, while the angle between two cells fluctuates around ${\theta }_{ij}=\pi \pm {\delta }_{\theta }$. These interactions between cells are particularly strong in the collision zone where there is a forwards flow of cells due to epithelial wetting and a backwards flow of cells caused by epithelial de-wetting (Pajic-Lijakovic and Milivojevic 2022a). Cell head-on interactions trigger cell repolarisation accompanied by weakening of the AJs and FAs per cell (Roycroft and Mayor 2016). In overcrowded surroundings under high cell compressive stress, cell repolarisation can be prolonged or even frozen. In this case, cells undergo jamming, i.e. the contractile-to-non contractile cell state transition.

The weakening and even detachment of AJs and FAs under cell mechanical stress causes energy dissipation leading to a decrease in the cell stress itself. Consequently, by regulating the strengths of AJs and FAs, cells can regulate wetting/de-wetting, epithelial viscoelasticity and their surface characteristics.

## Conclusion

Our theoretical analysis has examined the significance of cell–cell positional and orientational interactions in an overcrowded environment, leading to the emergence of various cell rearrangement scenarios, including (1) the epithelial-to-mesenchymal transition, (2) live cell extrusion, and (3) the cell jamming state transition. These scenarios share the common feature of being triggered by an accumulation of compressive stress within epithelial monolayers, coupled with an increase in cell packing density. The variety of possible cell responses suggests that another factor plays a role in regulating cell rearrangement. This factor is the cell shear stress, which can arise from either of two mechanisms: natural or forced convection. Natural convection, characterized by passive cell movement, is triggered by the interfacial tension gradient, whereas forced convection is a result of collective cell migration. The distribution of free energy in cell**–**cell and cell–matrix adhesion contacts affects the level of cell shear stress generated, consequently influencing the state of these adhesion contacts.

While cells can withstand tensional/compressive stress levels of a few kPa, shear stress in the range of a few tens of Pa, as observed during collective cell migration, can induce the formation of topological defects in cell alignment by promoting cell–cell orientational interactions. On the other hand, shear stress below 1 Pa may lead to the disruption of cell–cell adhesion, and even provoke the epithelial-to-mesenchymal transition. Unlike epithelial cells, mesenchymal cells respond to mechanical stress by enhancing their migratory behaviour.

Even if cells maintain their cell–cell adhesion contacts, disturbance in cell alignment can cause individual cell rotation, resulting in the generation of torsional stress between the cell and the extracellular matrix, which has the potential to rupture the focal adhesion. Despite this, the cells still maintain their epithelial phenotype and remain in an actively contractile state. To ensure homeostasis, specific cells that have lost their focal adhesions may be expelled, leading to a reduction in cell packing density and an increase in compressive stress within the cell population.

Cell jamming is induced by cell head-on interactions, which are efficient enough to trigger cell repolarisation accompanied by weakening of the cell–cell and cell–matrix adhesion contacts. An overcrowded environment is capable of damping the process of cell repolarisation by leading to cell contractile-to-noncontractile transition (i.e., the cell jamming). Despite this transition, the cells maintain their epithelial phenotype. The weakening of adhesion contacts between cells and the extracellular matrix leads to the dissipation of energy and a reduction in the mechanical stress experienced by the cells. Consequently, the cells undergo an unjamming transition, allowing them to resume their migration.

## Data Availability

Not applicable. No new data were created in the present research.
